# Use of complementary electrocardiogram-gated cardiac computed tomography in the assessment of aortic valve repairability: a pilot study^†^

**DOI:** 10.1093/icvts/ivaf086

**Published:** 2025-04-15

**Authors:** Valentina Mescola, Virginia Álvarez Asiaín, Javier De Diego Candela, Adela Navarro Echeverría, Félix Gomez Blasco, Facundo Machado Fernandez, Luis Jiménez Alfaro, Rafael Sádaba

**Affiliations:** Department of Cardiovascular Surgery, Navarra University Hospital, Pamplona, Spain; Department of Cardiovascular Surgery, Hospital Universitari Germans Trias i Pujol, Badalona, Spain; Department of Cardiology, Navarra University Hospital, Pamplona, Spain; IdiSNA, Navarra Institute for Healt Research, Pamplona, Spain; Department of Cardiovascular Surgery, Navarra University Hospital, Pamplona, Spain; Department of Cardiology, Navarra University Hospital, Pamplona, Spain; IdiSNA, Navarra Institute for Healt Research, Pamplona, Spain; Department of Cardiovascular Surgery, Navarra University Hospital, Pamplona, Spain; Department of Cardiovascular Surgery, Navarra University Hospital, Pamplona, Spain; Department of Cardiovascular Surgery, Navarra University Hospital, Pamplona, Spain; Department of Cardiovascular Surgery, Navarra University Hospital, Pamplona, Spain; IdiSNA, Navarra Institute for Healt Research, Pamplona, Spain

**Keywords:** aortic regurgitation, aortic valve repair, preoperative assessment, ECG-gated CT scan, multimodal imaging

## Abstract

**OBJECTIVES:**

In young patients with type I/II aortic regurgitation, the feasibility of aortic valve (AV) repair has been traditionally evaluated with echocardiography. We evaluate electrocardiogram (ECG)-gated cardiac computed tomography as a complement to transoesophageal echocardiography in the preoperative assessment of aortic repairability.

**METHODS:**

Patients undergoing non-urgent AV repair with or without aortic root replacement from October 2016 to May 2023 who had both imaging tests for the evaluation of AV repairability were included. The measurements obtained with echocardiography and ECG-gated scan of geometric height, aortic annulus and commissural orientation were compared using the intraclass correlation coefficient for inter-rater reliability.

**RESULTS:**

All 24 patients were males, with a median age of 47.5 years (41.9–59.3). Of these, 87.5% presented severe aortic regurgitation; 62.5% had associated aortopathy. 58.3% were in New York Heart Association class I and the remnant 41.7% in class II. The AV was bicuspid in 62.5% of the patients. The intraclass correlation coefficient indicated excellent reliability of ECG-gated scan for commissural orientation (ICC = 0.98, 95% CI: 0.96–0.99) and conjoined cusp geometric height (ICC = 0.91, 95% CI: 0.74–0.97) in bicuspid AVs, while in tricuspid valves it showed good reliability for left cusp geometric height (ICC = 0.76, 95% CI: 0.19–0.93) and annular diameter (ICC = 0.8, 95% CI: 0.33–0.95).

**CONCLUSIONS:**

ECG-gated cardiac scan complements echocardiography in aortic repair, showing excellent agreement for morphologic evaluation and commissural orientation. It is particularly reliable for geometric height in bicuspid valves and annular diameter and left cusp geometric height in tricuspid valves.

## GRAPHICAL ABSTRACT

**Figure:**
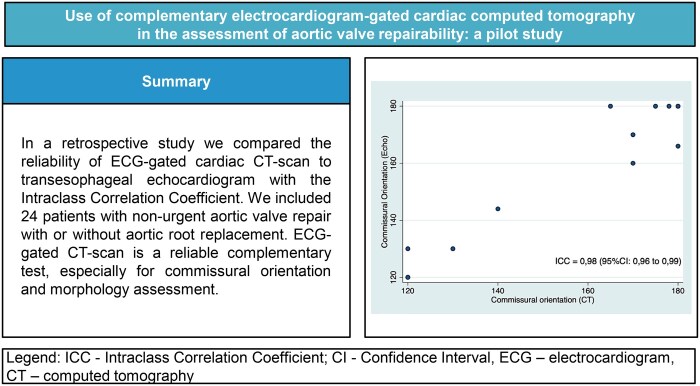

## INTRODUCTION

Most cases of chronic aortic regurgitation (AR) in high-income countries are due to degenerative valve disease and bicuspid aortopathy [1].

According to the El Khoury functional classification, aortic valve (AV) regurgitation can result from root or ascending aorta dilation with normal cusp motion (type I), or it can due to an abnormal cusp movement, such as in the presence of leaflet prolapse (type II) or restriction (type III) [2, 3].

AV repair represents a low-risk procedure with excellent long-term results when carried out in specialized and comprehensive valvular Heart Centres [4]. According to the 2021 European Society for Cardiology (ESC)/European Association for CardioThoracic Surgery (EACTS) Guidelines for the management of valvular heart disease, in young patients with type I or II AR in whom durable results are expected, the feasibility of AV repair should be considered [1, 4, 5].

In order to evaluate the feasibility of valve-sparing procedures, it is mandatory to carry out an exhaustive morphologic and functional assessment detailing all the parameters shown in Table 1 [6].

**Table 1:** Aortic valve parameters

Parameter	Definition	Values
Morphology	Depending on the number of cusps (based on the number of functional commissures)	Tricuspid: three fully developed commissures
		Bicuspid: two fully developed commissures and 0 or 1 raphe on the conjoined cusp
		Unicuspid: one fully developed commissure and two raphes
		Quadricuspid: four developed commissures
Commissural orientation	The angle formed by the lines joining the commissures to the center of the valve	Varies between 120° and 180°
Aortic annulus	Diameter of the virtual basal ring, measured on the plane that passes through the nadirs of the leaflets	18–23 mm
Geometric height (gH)	Distance from the nadir at the virtual basal ring to the free margin measured along the cusp	>16 mm (tricuspid AV) or >19 mm (non-conjoined cusp of BAV) for non-retracted cusps
Effective height (eH)	The orthogonal distance from the virtual basal ring to the free margin of the cusp	Around 9 mm (adults)
Coaptation height (cH)	The length of cusp apposition in diastole	4–5 mm

Definition and normal values.

Traditionally, these measurements, and therefore the possibility of AV repair, have been evaluated with echocardiography, especially with the transoesophageal modality (TOE) [2, 6–8].

The aim of our study was to evaluate if electrocardiogram (ECG)-gated cardiac computed tomography scan (ECG-gated CT) can be a useful complementary preoperative test, offering reliable measurements of the aortic annular diameter, the commissural orientation, and the geometric height (gH) in patients with significant AR or aortic dilation causing AR, compared to TOE.

## MATERIALS AND METHODS

### Ethics statement

This study was reviewed and approved by the Navarra University Hospital Ethic Committee (55–2018) and each patient signed an individual written consent. Data storage is consistent with the WMA Declaration of Taipei requirements.

### Data sources and study population

An institutional surgical database was reviewed to identify all patients above the age of 18 who underwent AV repair, with or without aortic root replacement, between October 2016 and May 2023, and who preoperatively had both TOE and ECG-gated CT for the evaluation of AV repairability. The patients were referred to surgery due to significative, mostly severe, symptomatic AR and/or significative aortic dilation causing AR.

Patients with acute aortic syndrome and/or emergent procedures were excluded.

The data were extracted by two reviewers working in consensus.

### Outcomes

The primary outcome of the study was to evaluate the reliability of ECG-gated CT compared to TOE in the preoperative analysis of the aortic annulus, the gH and the commissural orientation.

The secondary end-points of the study were early postoperative outcomes, such as mortality, stroke, success of repair, echocardiographic valvular assessment pre-discharge and clinical postoperative status.

### Variables

Patients’ demographics and comorbidities were obtained from electronical medical record.

Aortopathy was defined as the presence of aneurysms or dilation in any segment of the entire aorta, from the root to the descending portion. High blood pressure was defined according to the 2018 ESC/European Society of Hypertension (ESH) Clinical Practice Guidelines for the Management of Arterial Hypertension [9].

Preoperative clinical presentation, including the New York Heart Association (NYHA) status, was evaluated by cardiologists of the Aortic Team.

All patients underwent comprehensive TOE using an EPIQ CVx-3d (Philips, Netherlands) ultrasound system. Post-processing was performed using dedicated software (QLAb Version 9.0, 3DQ Advanced Software, Philips, Netherland, and TomTec 4DLV analysis software 2.7 Image-Arena version 4.1; Build 4.1.1.30, TomTec, Germany) to create a multiplanar reconstruction.

AV morphology and dysfunction assessment was based on the parameters shown in Table 1, motion and jet characteristics, combining the midoesophageal long- and short-axis and the deep transgastric views [7, 10].

The mechanism of regurgitation was classified according to the El Khoury functional criteria and graded from 0 to 4 following the European Association of Cardiovascular Imaging (EACVI)/ESC recommendations [11].

Trans-aortic gradients were recorded at rest and they were calculated using the modified Bernoulli equation.

Volumetric measurements followed the European Society of Echocardiography (ESE) recommendations [7, 12].

Preoperative ECG-gated CT was performed with a Brillance 64 CT scanner (Philips, Netherlands), with cardiac gating in fully retrospective mode. The studies were performed at 120 kV in order to reduce overall radiation dose. Intravenous beta-blockers were administrated to patients without contraindications and heart rates above 80 beats per minute [13].

CT data sets were post-processed using dedicated software (Intellispace portal, Phillips, Netherlands) for multiplanar and three-dimensional reconstruction.

Measurements included the AV, aorta and the coronary tree.

The feasibility of the AV repair was assessed by evaluating the mechanism of regurgitation, focusing especially on [2, 7, 10, 12, 14, 15]:

- The presence of annular dilation that could be restored with an annuloplasty technique.
- The absence of cusp retraction, to guarantee the presence of sufficient leaflet tissue to ensure a correct post repair effective height (eH) and coaptation height (cH).
- The symmetry of the leaflets, suggested by commissural orientation close to 120° and 180° for tricuspid and bicuspid valves (BAV), respectively.

**Figure 1:**
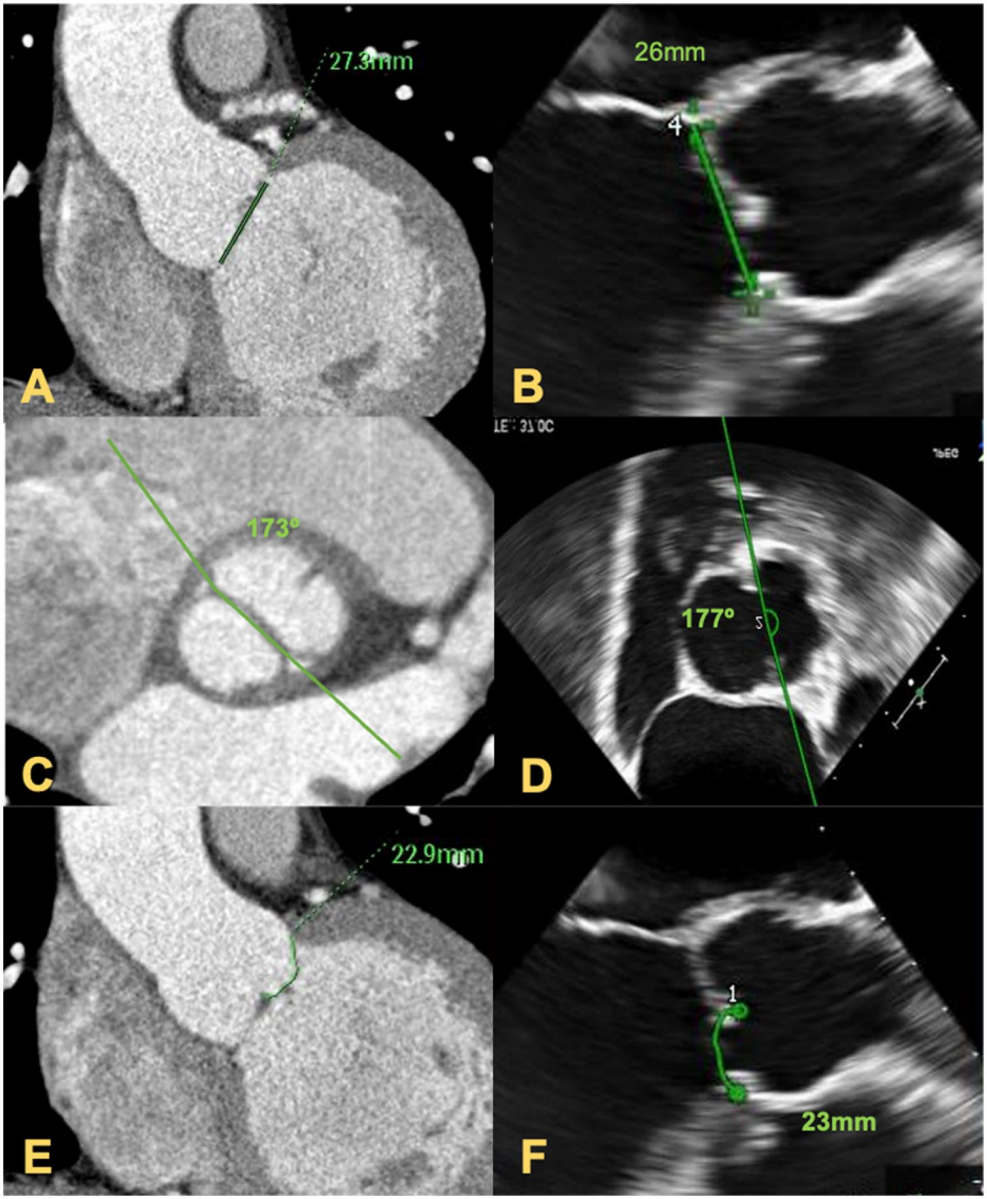
Comparison of TOE and ECG-gated CT scan images. Assessment of aortic repairability with TOE and ECG-gated CT. The virtual basal ring is measured in the same patient using CT scan (**A**) and TOE (**B**), as showed by the mark. Comparison of the commissural orientation (green) in a bicuspid patient using CT (**C**) and TOE (**D**). Measurements of the conjoined cusp geometric height in a bicuspid patient with CT (**E**) and TOE (**F**), marked

Then, the following measurements of the AV obtained with the TOE and ECG-gated CT were compared for each patient (Fig. 1):

- Aortic annulus diameter (mm).
- gH (mm).
- Commissural orientation (degrees, °).

### Statistical analysis

The inter-rater reliability or homogeneity of measurements of the two different imaging techniques was assessed using the intraclass correlation coefficient (ICC). Reliability value ranges between 0 and 1, with values closer to 1 representing stronger reliability [16].

The ICC estimate was calculated on a single rating, consistency of agreement, two-way random effects model using Stata 16 (StataCorp. 2019. Stata Statistical Software: Release 16. College Station, TX: StataCorp LLC).

Based on the 95% confident interval of the ICC estimate, values less than 0.5, between 0.5 and 0.75, between 0.75 and 0.9, and greater than 0.90 are indicative of poor, moderate, good and excellent reliability, respectively [17].

In order to reduce the risk of Type I errors in multiple comparisons, we adjusted the significance level according to the Bonferroni method, considering the significance of *P-*value for each test equal to its alpha (0.05) divided by the number of tests performed. Seven hypotheses were adjusted (commissural orientation, annular diameter, conjoined gH and non-conjoined gH in BAV, left gH, right gH and non-coronary gH in TAV) and the significance level set to 0.007.

The kappa method was used for assessing agreement in dichotomous variables [18].

## RESULTS

After reviewing the institutional database, we identified 24 adult patients who preoperatively had both TOE and ECG-gated CT and that underwent AV repair with or without aortic root replacement between October 2016 and May 2023.

The patients were all males and baseline characteristics are summarized in Table 2.

**Table 2:** Preoperative clinic and echocardiographic characteristics

Baseline characteristics	
Age (years)	47.5 (41.9–59.3)
Weight (kg)	78 (9)
Height (m)	1.8 (0.11)
Male gender	100.00 (24)
Comorbidities	
Hypertension^a^	58.3 (14)
Aortopathy^b^	62.5 (15)
Significative Coronay Disease^c^	4.2 (1)
Clinical status	
NYHA class I	58.3 (14)
NYHA class II	41.7 (10)
Mitral regurgitation	
None	62.5 (15)
Mild	37.5 (9)
Morphology	
Tricuspid aortic valve	37.5 (9)
Bicuspid aortic valve	62.5 (15)
Bicuspid type 0	13.3 (2)
Bicuspid type 1	86.7 (13)
El Khoury functional classification	
Type I	33.3 (8)
Type II	66.7 (16)
Type III	0 (0)
Severity of aortic regurgitation	
I	4.2 (1)
II	8.3 (2)
III	54.2 (13)
IV	33.3 (8)
LVEF impairement	
None	87.5 (21)
Mild	8.3 (2)
Moderate	4.2 (1)
Severe	0 (0)
Trans-aortic gradients	
Mean trans-aortic gradient (mmHg)	1.5 (7.05)
Maximum trans-aortic gradient (mmHg)	2.75 (3.8)

Continuous variables are expressed in median (interquantile range), while categorical ones are reported with the percentage (absolute number of observations). Age is reported as median and interquantile range in years.

a Defined as systolic values ≥140 mmHg and/or diastolic blood pressure values ≥90 mmHg.

b Presence of aneurysms or dilation in any segment of the aorta.

c Coronary stenosis ≥70%.

The most common comorbidities were hypertension (58.3%) and associated aortopathy (62.5%).

All patients referred some degree of dyspnoea on exertion, and most presented severe AR (87.5%), except for one patient presenting mild and two presenting moderate AR. 37.5% of patients had a tricuspid AV (TAV) and the remnant 62.5% a bicuspid one (type 0 in two patients, type 1 in 13 patients).

**Figure 2:**
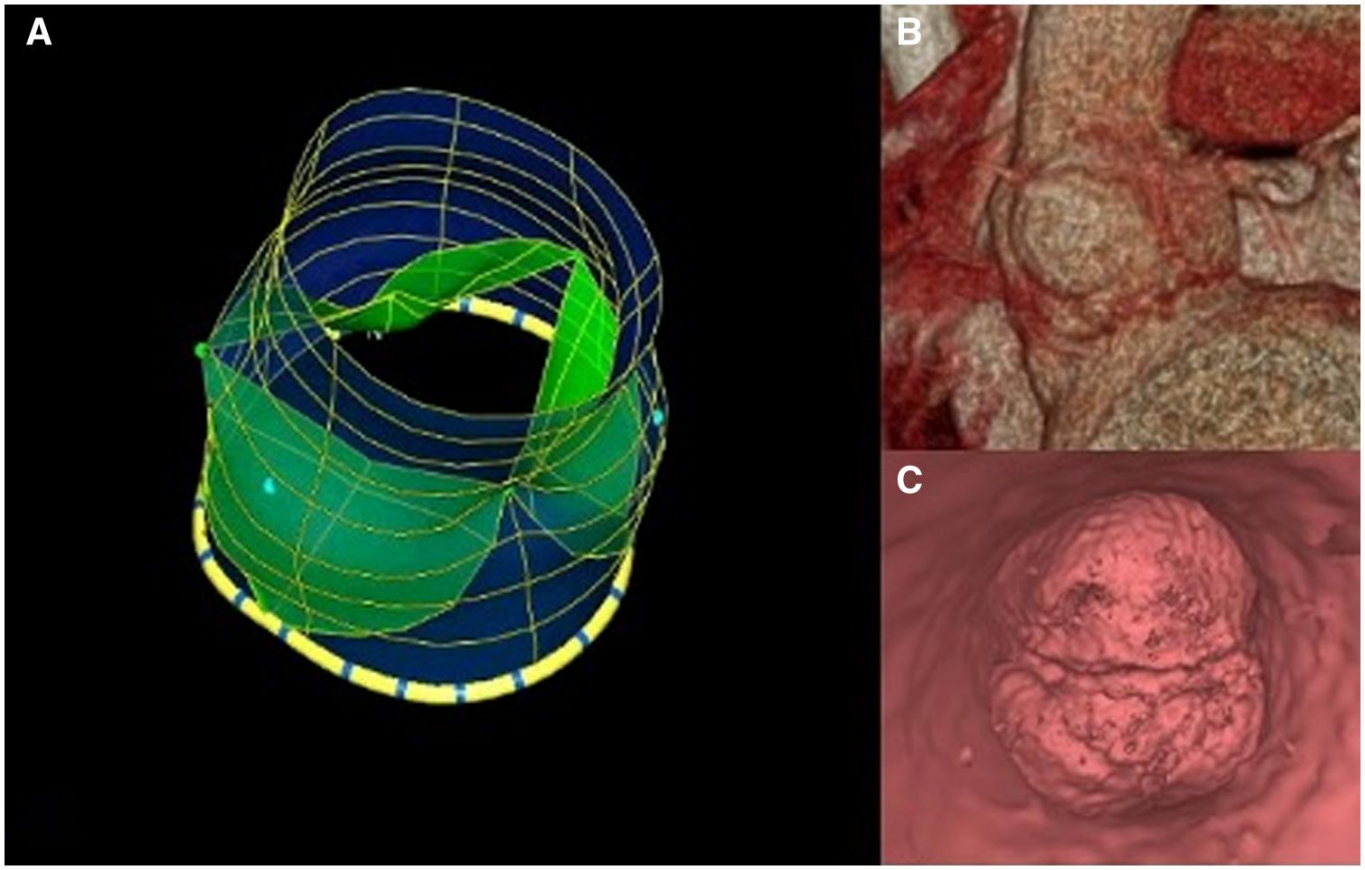
Three-dimensional reconstruction with echocardiography and CT scan. Multiplanar reconstruction using both TOE and CT scan. (**A**) Aortic root echocardiographic three-dimensional reconstruction. (**B**) Volume-rendering processed image of the aortic root proceeding from ECG-gated cardiac CT scan. (**C**) AV endoscopic view acquired through CT scan

Additionally, measurements of different parts of the aortic root and aorta were taken using both TOE and ECG-gated CT (Table 3, Fig. 2).

**Table 3:** Echocardiographic and ECG-gated CT scan preoperative assessment

	Median	IQR	Min	Max
Measurements in bicuspid valves (TOE)				
Virtual basal ring (mm)	28.0	5.0	24	32
Sinotubular junction (mm)	32.0	12.0	24	43
Ascending aorta (mm)	39.0	10.0	25	48
Geometric height conjoined cusp (mm)	21.0	4.0	14	33
Geometric height non-conjoined cusp (mm)	21.0	2.0	18	23
Coaptation height (mm)	5.0	2.5	3	8
Effective height (mm)	9.0	1.5	6.5	13
Commissural orientation (°)	180.0	10.0	144	180
Measurements in tricuspid valves (TOE)				
Virtual basal ring (mm)	28.0	2.0	25	36
Sinotubular junction (mm)	41.0	8.0	28	49
Ascending aorta (mm)	41.0	11.0	35	59
Geometric height left cusp (mm)	20.0	1.0	18	25
Geometric height right cusp (mm)	21.0	3.0	17	23
Geometric height non-coronary cusp (mm)	21.0	2.0	19	23
Coaptation height (mm)	7.2	1.0	4	9.5
Effective height (mm)	14.0	1.5	5	15
Commissural orientation (°)	168.0	60.0	120	130
Measurements in bicuspid valves (CT)				
Virtual basal ring (mm)	30	5	22	34
Sinotubular junction (mm)	32	10	21	37
Ascending aorta (mm)	38	18	25	48
Geometric height conjoined cusp (mm)	22	3	19	34
Geometric height non-conjoined cusp (mm)	22	4	17	24
Coaptation height (mm)	4.6	2	3	7
Effective height (mm)	7	4.5	5	12
Commissural orientation (°)	180	10	134	180
Measurements in Tricuspid valves (CT)				
Virtual basal ring (mm)	29	4	25	35
Sinotubular junction (mm)	43	11	28	50
Ascending aorta (mm)	41	12	31	59
Geometric height left cusp (mm)	20	3	18	23
Geometric height right cusp (mm)	20	3	17	27
Geometric height non-coronary cusp (mm)	21	3	19	23
Coaptation height (mm)	5	1	4	12
Effective height (mm)	9	4	5	17
Commisural orientation (°)	120	0	120	130

Continuous variables are expressed as median and interquantile range (IQR). The minimum and maximum values are reported.

**Figure 3:**
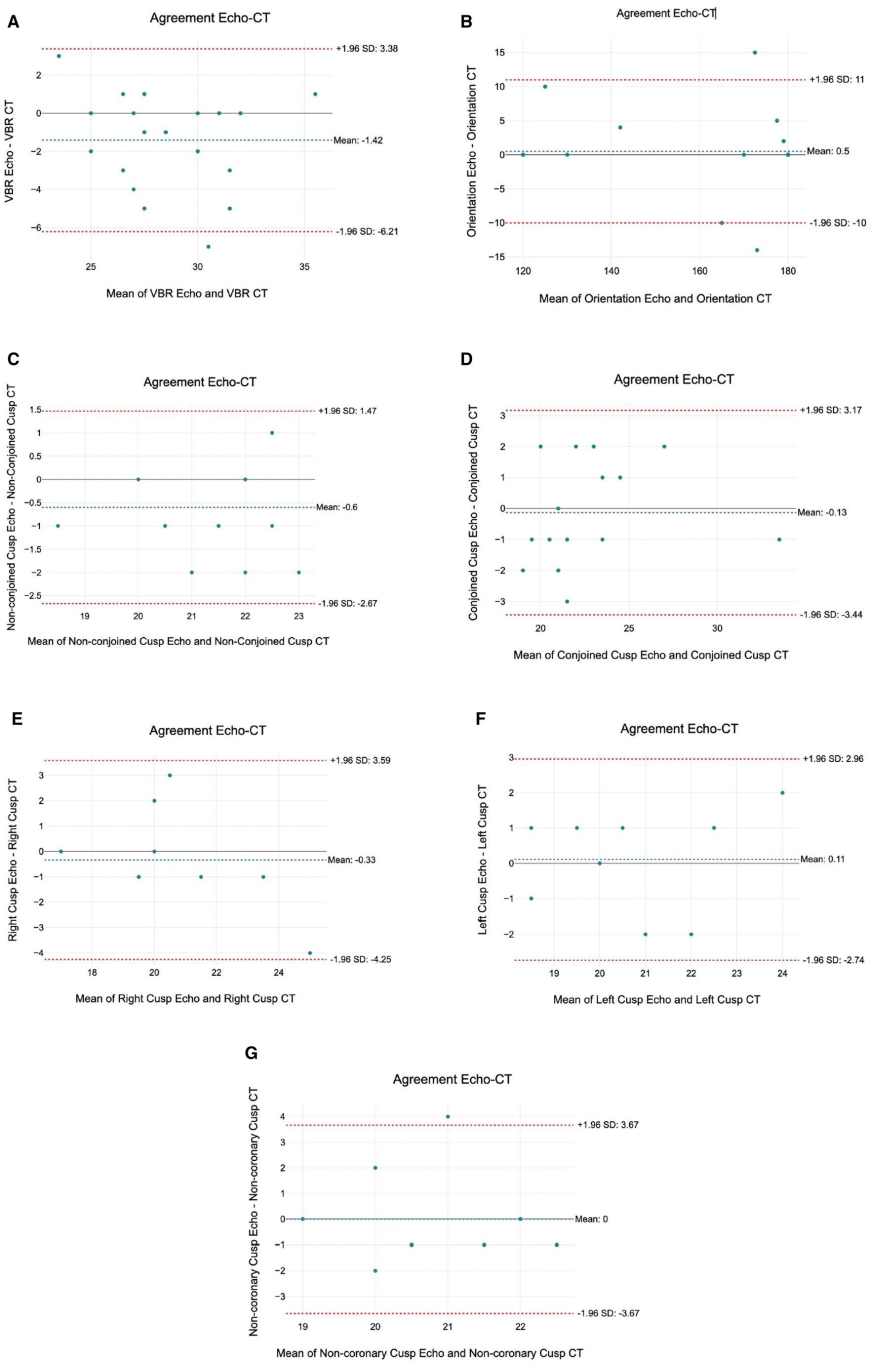
Bland–Altman plots for echo/CT agreement. Agreement for the virtual basal ring (**A**), the commissural orientation (**B**), the conjoined and non-conjoined cusps in BAV (**C**, **D**)

The ICC estimates for inter-rater reliability between TOE and CT measurements are depicted in Table 4, Fig. 3 and Supplementary Fig. S1:

**Table 4:** Intraclass correlation coefficient between TOE and ECG-gated CT

	ICC	CI limits	*P*
Bicuspid valves (*n* = 15)			
Geometric height conjoined cusp	0.91	0.74–0.97	0.000
Geometric height non-conjoined cusp	0.68	0.28–0.88	0.002
Virtual basal ring	0.59	0.12–0.84	0.009
Tricuspid valves (*n* = 9)			
Geometric height left cusp	0.76	0.19–0.93	0.008
Geometric height right cusp	0.70	0.11–0.92	0.013
Geometric height non-coronary cusp	0.15	−0.53 to 0.72	0.335
Virtual basal ring	0.80	0.33–0.95	0.003
Commissural orientation			
	0.98	0.96–0.99	0.000
Morphologic classification bi/tricuspid	*k*	SE	*P*
	1	0.20	0.000

Intraclass correlation coefficient (ICC) between TOE and ECG-gated CT with confidence interval limits (CI). Based on the 95% IC of the ICC estimate, values less than 0.5, between 0.5 and 0.75, between 0.75 and 0.9, and greater than 0.90 are indicative of poor, moderate, good and excellent reliability, respectively. As multiple comparisons are made, the significance of *P*-value is adjusted according to the Bonferroni method to 0.007. For the agreement of the morphologic classification into bicuspid and tricuspid valves the kappa method is used and reported.

- For gH in BAV, ICC indicated excellent reliability for the conjoined leaflet and good reliability for the non-conjoined one.
- For gH in TAV, ICC indicated good reliability for the left cusp, moderate reliability for the right cusp and poor reliability for the non-coronary cusp.
- For the aortic annulus, ICC showed good reliability in TAV and moderate reliability in BAV.
- For commissural orientation, the two techniques presented almost complete homogeneity.
- For morphologic classification in BAV and TAV, the agreement was 100% (*k* = 1).

Regarding the secondary end-points of the study, 23 patients out of 24 received a successful repair. One patient, instead, received a mechanical valve replacement due to the damage of the native valve during dissection.

In the postoperative period, none of the patients presented a stroke. Pre-discharge and 6 months follow-up TTE showed no residual AR in 13 patients and grade I AR in 10.

All the patients of the series are alive and in good functional class (NYHA class I).

## DISCUSSION

The Heart Team plays a crucial role in selecting suitable candidates for AV repair. In order to plan a successful repair, it is of paramount importance to understand the mechanism of AR [3].

The ideal candidate for repair is a young patient, with a low surgical risk and a good quality of life [1, 4].

Regarding the mechanism of AR, this should not be due to leaflet restriction (gH at least 16 mm for TAV, or 19 mm for BAV) [6, 19–21].

Echocardiography is the fundamental tool to properly evaluate the feasibility of repair. TOE, in particularly, represents a non-radiative and relatively harmless test that provides a dynamic evaluation of the AV dysfunction [8, 15].

But, as beautifully pointed out by Tretter and colleagues, we should advocate for the most complete way to fix the problem [19]. A multimodal imaging approach, rather than TOE or surgeon expertise alone, is advocated to improve valvular assessment and minimize discrepancies in preoperative measurements that, even when slight, can have a significant impact on the repair [6].

This helps the Heart Team in deciding the most appropriate manoeuvre, either in remodelling or reimplantation procedure, when an intervention on the leaflet is required (as central plication for prolapse) or otherwise to be avoided, as cusp retraction is a challenging substrate for repair [2, 22].

The combination of an ECG-gated CT reconstruction and TOE provides a comprehensive characterization of the leaflet abnormalities and helps to predict the quality and durability of the repair [12, 19, 23].

TOE offers real-time, dynamic evaluation of valve function and haemodynamic. It is, indeed, the most cost-effective technique, but depends on the operator expertise and the adequacy of the acoustic windows [12].

ECG-gated CT scan, on the contrary, offers standardized imaging acquisition, allowing non-subspecialized professionals to perform the scan for later evaluation by the Aortic Team.

Moreover, while TOE is usually limited to the proximal aorta, the CT scan provides information on the entire aorta, coronary *ostia* position and their relation to the commissures (Fig. 2) [19, 23].

So, CT scan helps in choosing the best access or cannulation site (particularly important for the mininvasive or redo approach), informs on concomitant coronary or thoracic disease and complements other imaging techniques in understanding the complex relationship between the aortic root and left and right ventricle outflow tract [24, 25].

The integration of different imaging modalities is crucial for comprehensive postoperative surveillance [19]. While echocardiography is suitable for monitoring valve function, periodic CT scans are often necessary to assess the remodelling of valve-sparing procedures and evaluate the remaining aorta [19].

As we show, CT reliably measures the key predictors of repair (annular dilation, the symmetry of the leaflets and the presence of adequate leaflet tissue [26]), in addition to the usual data on thoracic and aortic anatomy.

In our series, the two imaging techniques showed perfect concordance in morphological classification and almost perfect homogeneity in measuring the commissural orientation [14]. This parameter is particularly important, especially in BAV, as the increased commissural asymmetry often indicates potential complexity in the repair [26].

The accuracy of both the morphologic classification and the commissural orientation was confirmed intraoperatively.

Regarding the moderate reliability for the ‘annular’ diameter in BAV, we must consider its dynamic nature, becoming elliptical in diastole and nearly circular in systole. TOE measures the annulus in the long axis on an oblique plane, while, CT does it in the short-axis [19]. This, and the asymmetry of the bicuspid annulus, can explain the lower ICC for the virtual basal ring in BAV [24].

Regarding the gH reliability, in BAV, CT scan showed excellent reliability for the conjoined cusp, which is typically larger than the non-conjoined leaflet. For tricuspid valves, left and right cusps were reliably measured with CT scan, while the ICC for non-coronary cusp showed poor reliability due to a significant discrepancy in subject number 17 caused by movement artefact affecting the correct measurement.

It is mandatory to acknowledge the several limitations this pilot study presents.

First, as a retrospective single-centre study, the study’s findings may have restricted generalizability to broader populations. Second, the observational design precludes establishing definitive causal relationship.

The small cohort size represents a critical limitation, as any measurement discrepancies between techniques can substantially impact overall concordance. Consequently, more comprehensive, prospective studies with larger sample sizes are needed to validate these initial findings.

Moreover, although the evidence on multimodal evaluation before valve surgery is wide and growing, we lack of studies comparing the two techniques in the measurements we focused on. Due to the forementioned and to the significant results obtained, the series has been considered a pilot study and no power calculation was performed.

An additional complexity stems from the extended study period spanning 2016–2023. The Aortic Valve Repair Program’s developmental trajectory introduces potential variability. Initially launched in late 2016, the program did not fully mature until the dedicated Aortic Unit’s establishment in 2021, post-pandemic. Of the 24 patients studied, only nine were treated between 2016 and 2021, meaning early results could be significantly influenced by the professional team’s learning curve. A total of 27 patients underwent aortic repair during the study period.

Moreover, radiation exposure and renal complications are, indeed, potential risks associated with ECG-gated cardiac CT scans. We implemented strategic technical and procedural modifications, in order to reduce overall patient risk.

Radiation dose management was achieved through multiple technical interventions. The scans were performed at a low 120 kV, with sophisticated ECG dose modulation techniques that selectively reduce milliamperage during non-critical imaging intervals. Scanning was typically confined to a single cardiac beat, with exceptions made only for patients exhibiting high heart rates requiring multisegmented reconstruction [13].

Contrast-related risks were carefully managed through strategic timing. All patients underwent non-urgent operations, and the preoperative tests (including the ECG-gated CT scan) were systematically performed before discussing the patient in the Heart Team, allowing for more than one month between CT scanning and surgical intervention. This prudent interval provided ample time for potential renal recovery. Furthermore, no patients with pre-existing significant renal dysfunction received an ECG-gated CT scan.

In our experience, the use of CT scans in the preoperative evaluation for aortic repair appears to be a safe and valuable tool that does not add significant risk and cost. More than a half of the patients in the sample would have undergone CT scans due to concomitant aortopathy in any case. For patients with a low probability of coronary artery disease, the option of using a non-invasive coronarography is already discussed in the last ESC/EACTS Guidelines on valvular heart disease, and recent data confirm CT as a safe and reliable alternative before valve surgery [1, 27].

## CONCLUSIONS

In our experience, ECG-gated CT scan complements TOE in the evaluation of the AV repairability, resulting in an additional coherent test. ECG-gated CT scan, to TOE, showed excellent agreement in the morphology evaluation and commissural orientation, and it is particularly reliable when measuring gH in BAV, and annulus and left gH in TAV.

## Supplementary Material

ivaf086_Supplementary_Data

## Data Availability

The data underlying this article will be shared on reasonable request to the corresponding author.

## ABBREVIATIONS

AR: Aortic regurgitation
AV: Aortic valve
BAV: Bicuspid aortic valve
cH: Coaptation height
ECG-gated CT: Electrocardiogram-gated cardiac computed tomography scan
eH: Effective height
gH: Geometric height
ICC: Intraclass correlation coefficient
LVEF: Left ventricular ejection fraction
NYHA: New York Heart Association
TAV: Tricuspid aortic valve
TOE: Transesophageal echocardiography
