# Supplementary figures and images for: Use of complementary electrocardiogram-gated cardiac computed tomography in the assessment of aortic valve repairability: a pilot study
Source: Interdiscip Cardiovasc Thorac Surg. 2025 Apr 15;40(4):ivaf086. doi: 10.1093/icvts/ivaf086 (PMC12043003; doi:10.1093/icvts/ivaf086)

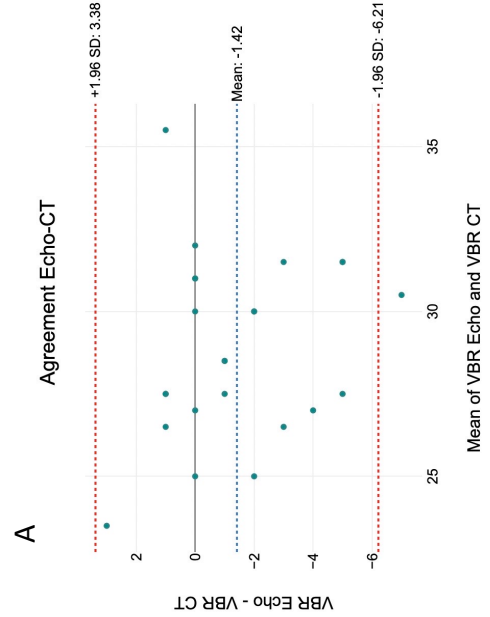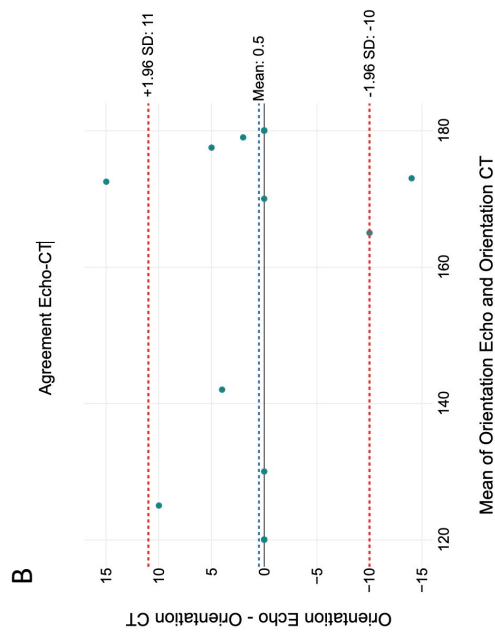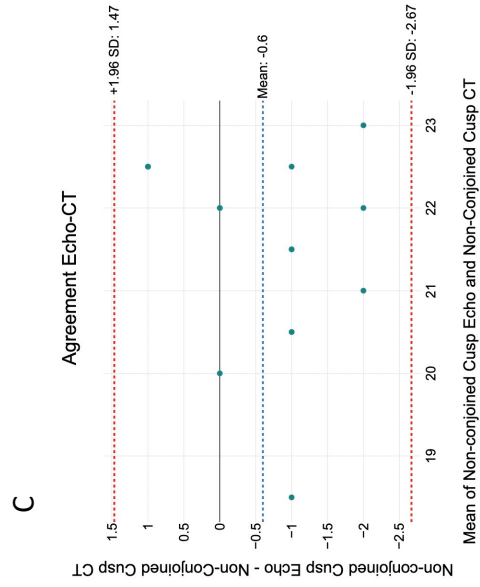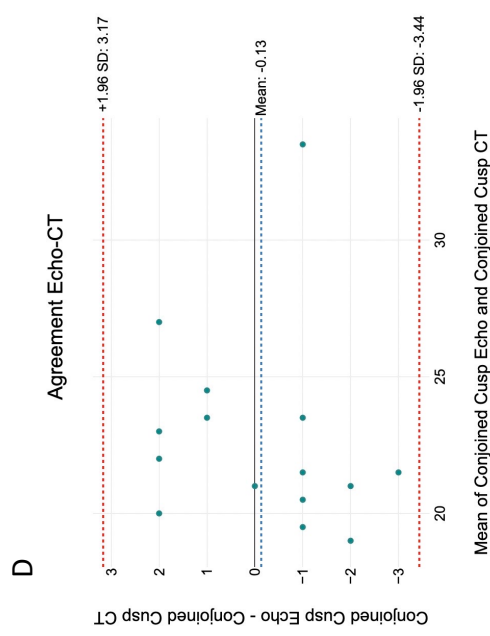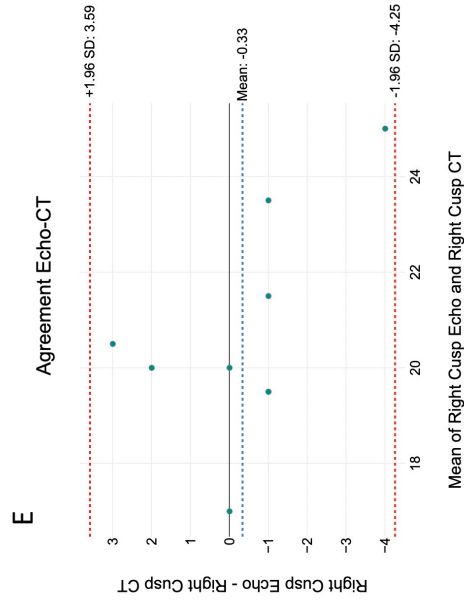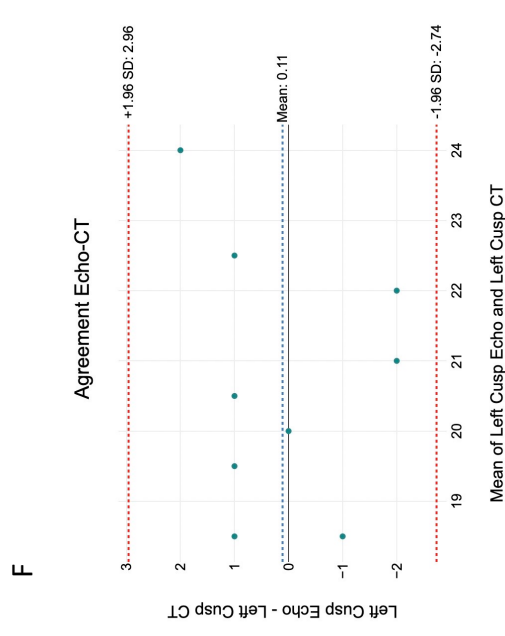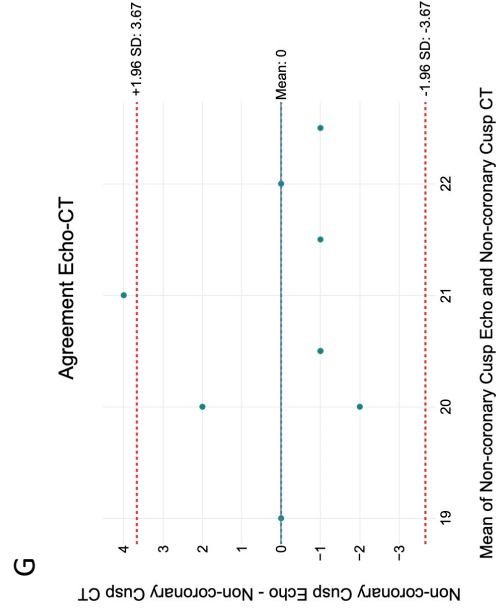

Supplement: ivaf086_Supplementary_Data [file ivaf086_supplementary_data.zip › B-A.pdf]
