# Supplementary material for: Use of complementary electrocardiogram-gated cardiac computed tomography in the assessment of aortic valve repairability: a pilot study
Source: Interdiscip Cardiovasc Thorac Surg. 2025 Apr 15;40(4):ivaf086. doi: 10.1093/icvts/ivaf086 (PMC12043003; doi:10.1093/icvts/ivaf086)
Supplement: ivaf086_Supplementary_Data [file ivaf086_supplementary_data.zip › ivaf086_Supplementary_Data.zip!/Supplementary Figure Legend.docx]

**Supplementary Figure Legend**

**Supplementary Fig 1. Bland-Altman plots for Echo/CT agreement.** Agreement for right (E), left (F) and non-coronary (G) cusps in TAV.
